# Retrospective Screening of Clinical Samples for Monkeypox Virus DNA, California, USA, 2022

**DOI:** 10.3201/eid2904.221576

**Published:** 2023-04

**Authors:** Caitlin A. Contag, Jacky Lu, Zachary T. Renfro, Abraar Karan, Jorge L. Salinas, Michelle Khan, Daniel Solis, Malaya K. Sahoo, Fumiko Yamamoto, Benjamin A. Pinsky

**Affiliations:** Stanford University School of Medicine, Stanford, California, USA

**Keywords:** mpox, monkeypox virus, viruses, sexually transmitted infections, zoonoses, orthopoxvirus, chlamydia, gonorrhea, syphilis, human immunodeficiency virus, United States

## Abstract

We retrospectively screened oropharyngeal and rectal swab samples originally collected in California, USA, for *Chlamydia trachomatis* and *Neisseria gonorrhoeae* testing for the presence of monkeypox virus DNA. Among 206 patients screened, 17 (8%) had samples with detectable viral DNA. Monkeypox virus testing from mucosal sites should be considered for at-risk patients.

Monkeypox virus (MPXV) is an enveloped, double-stranded DNA virus in the family *Poxvirus*, genus *Orthopoxvirus*, and is related to variola, the causative agent of smallpox. In 2022, MPXV transmission caused a large global mpox disease outbreak that disproportionately affected male persons who identified as gay, bisexual, and men who have sex with men (MSM) and persons who identified as transgender (*1*). The clinical manifestations of MPXV infection also evolved from prior outbreaks; more patients in 2022 had anogenital rash and proctitis, rather than disseminated cutaneous lesions (*1*). During prior mpox outbreaks, asymptomatic or subclinical MPXV infection was thought to be rare, but evidence from the 2022 outbreak suggests that infected patients can have minimal symptoms (*2*,*3*). To identify persons with subclinical MPXV infection, we retrospectively analyzed oropharyngeal and rectal swab samples submitted for *Chlamydia trachomatis* and *Neisseria gonorrhoeae* (CT/NG) testing at a tertiary academic medical center.

Swab samples were collected at Stanford Health Care by using the Aptima Multitest Swab Specimen Collection Kit for the Aptima Combo 2 Assay (Hologic, https://www.hologic.com). We included all samples collected during July 7–September 6, 2022 that had sufficient residual volume. The study was approved by the Stanford University institutional review board (protocol no. 66786).

We extracted total nucleic acids from 300 μL of Aptima Specimen Transport Medium (Hologic) by using the Chemagic instrument (PerkinElmer, https://www.perkinelmer.com), according to the manufacturer’s recommendations. To test for MPXV DNA, we used 2 laboratory-developed quantitative PCR (qPCR) assays modified from Centers for Disease Control and Prevention published assays (*4*,*5*). The first qPCR targeted viral DNA polymerase sequence conserved throughout nonvariola orthopoxviruses, including MPXV. The second qPCR targeted the viral tumor necrosis factor (TNF) receptor sequence specific for MPXV clade II (formerly West African clade). We performed qPCR reactions as previously described (*6*), except we used the CFX96 thermal cycler (Bio-Rad, https://www.bio-rad.com). We tested all specimens with both qPCR assays and interpreted samples with concordant MPXV as mpox-positive and samples without detected MPXV as mpox-negative. When there was discordance between viral DNA polymerase and the viral TNF receptor targets, we repeated both reactions from the eluate and interpreted the sample as positive only if MPXV was reproducibly detected. We excluded 3 concordant negative samples in which the internal control (β-globin gene) failed in one or both reactions.

A total of 347 swab samples submitted for CT/NG testing from 206 patients met the inclusion criteria: 195 (56%) oropharyngeal and 152 (44%) rectal swab specimens. Patients ranged in age from 7 days–77 years (mean 35 years). Most (176/206; 85%) patients were male; 1 patient was assigned male at birth but identified as genderqueer. Twelve patients in this cohort had known MPXV infection diagnosed via lesion qPCR.

Overall, we detected viral DNA in 24/347 (7%) samples, including 11/195 oropharyngeal and 13/152 rectal swab specimens, representing 17/206 (8%) patients (Figure). Among 17 patients who tested MPXV-positive, 6 (35%) had received an mpox vaccine. No patients received a vaccine >14 (range 4–12) days before their positive test. Of 12 persons with known mpox who underwent CT/NG testing, 11 (91.7%) had detectable MPXV DNA from swab samples, comprising 16 positive samples. Six patients without lesions or diagnosed mpox had detectable MPXV DNA from CT/NG swabs, comprising 8 positive samples (Table). Of those patients, 3 (50%) were asymptomatic and 3 (50%) symptomatic; 2 had proctitis and 1 had pharyngitis. Like patients with known mpox, all 6 patients with newly diagnosed mpox were MSM. In addition, 50% (3/6) of patients with new mpox diagnoses were co-infected with another sexually transmitted infection compared with 42% (5/12) of patients with known MPXV infections, 33% (2/6) were HIV-positive (vs. 42%; 5/12), and 33% (2/6) were on HIV preexposure prophylaxis (vs. 58%, 7/12).

**Figure:**
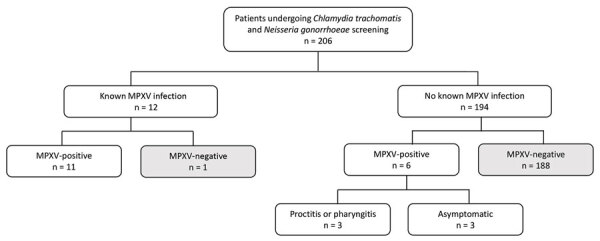
Flowchart of retrospective quantitative PCR screening for MPXV in oropharyngeal and rectal swab samples submitted for *Chlamydia trachomatis* and *Neisseria gonorrhoeae* testing, California, USA, 2022. Patients with known MPXV infection (n = 12) were diagnosed by quantitative PCR of cutaneous lesions. Patients without known mpox did not have MPXV-positive tests or cutaneous lesions at the time of specimen collection for *C. trachomatis* and *N. gonorrhoeae* testing. MPXV, monkeypox virus.

**Table:** Patients with newly diagnosed mpox via retrospective qPCR of oropharyngeal or rectal swab samples, California, USA, 2022*

Age, y/sex	Sample type, Ct values†		Clinical manifestations and history	History of HIV or PrEP	Concurrent STI
	Oropharyngeal	Rectal			
55/M	ND/ND	28.6/29.4	Asymptomatic; sought CT/NG testing after notification of STI exposure 2 wks before at a sex club in Europe; specific sexual practice unknown	PrEP	Syphilis
30/M	ND/ND	35.5/35.1	Asymptomatic; undergoing routine CT/NG screening; exposure unknown	PrEP	None diagnosed
17/M	37.5/38.2	22.0/24.0	Asymptomatic; undergoing STI screening after recent vaginal sex and receptive and insertive oral and anal sex with male and female partners; timing of exposure unknown; MPXV-positive cutaneous lesions subsequently developed	HIV-1–negative; not on PrEP	Chlamydia, gonorrhea, syphilis
31/M	29.6/29.8	Not collected	Sore throat, tonsillar exudates, and lymphadenopathy 4 d after sexual encounter with a male partner 11 d before testing	Unknown	None diagnosed
29/M	ND/ND	17.2/17.9	Hematochezia; reported receptive anal sex 4 wk before testing	HIV-1–positive	None diagnosed
46/M	34.2/34.7	19.1/19.2	Hematochezia and rectal pain; reported recent receptive anal sex	HIV-1–positive	Chlamydia, gonorrhea
*Ct, cycle threshold; CT/NG, *Chlamydia trachomatis* and *Neisseria gonorrhoeae*; MPXV, monkeypox virus; ND, not detected; PrEP, preexposure prophylaxis; qPCR, quantitative PCR; STI, sexually transmitted infection. †Results indicate Ct for viral DNA polymerase/viral tumor necrosis factor receptor.					

Our findings demonstrate that patients without cutaneous lesions can be MPXV-positive, which is consistent with observations reported in similarly designed studies in Europe (*7*,*8*). Occult infection with oropharyngeal and rectal viral shedding might have contributed to the scale of the 2022 mpox outbreak, which spread through sexual networks. All but 1 patient with known MPXV infection in our cohort had detectable viral DNA in oropharyngeal or rectal swab samples, suggesting that the new infections we detected are likely true positives. Furthermore, all newly identified mpox patients in our study had >1 sample for which both qPCR targets were detected. 

Current MPXV tests cleared for emergency use are indicated only for use on lesion samples (*9*). Further studies are needed to characterize viral shedding dynamics, particularly related to symptom onset and duration of infectivity. As data demonstrating mucosal viral shedding in mpox emerge, expanding testing to allow broader sample collection and expedite diagnostic validation of samples from various anatomic sites will be crucial.

In conclusion, during the ongoing mpox outbreak, clinicians should consider oropharyngeal and rectal MPXV qPCR testing for at-risk patients with pharyngitis or proctitis. In addition, asymptomatic screening in high-risk populations might be warranted if community prevalence is high or rising. 

## References

[1] Thornhill JP, Barkati S, Walmsley S, Rockstroh J, Antinori A, Harrison LB, et al.; SHARE-net Clinical Group. Monkeypox virus infection in humans across 16 countries—April–June 2022. N Engl J Med. 2022;387:679–91. 10.1056/NEJMoa2207323 35866746

[2] Abbasi J. Reports of asymptomatic monkeypox suggest that, at the very least, some infections go unnoticed. JAMA. 2022;328:1023–5. 10.1001/jama.2022.15426 36044244

[3] Jezek Z, Marennikova SS, Mutumbo M, Nakano JH, Paluku KM, Szczeniowski M. Human monkeypox: a study of 2,510 contacts of 214 patients. J Infect Dis. 1986;154:551–5. 10.1093/infdis/154.4.551 3018091

[4] Li Y, Olson VA, Laue T, Laker MT, Damon IK. Detection of monkeypox virus with real-time PCR assays. J Clin Virol. 2006;36:194–203. 10.1016/j.jcv.2006.03.012 16731033 PMC9628957

[5] Li Y, Zhao H, Wilkins K, Hughes C, Damon IK. Real-time PCR assays for the specific detection of monkeypox virus West African and Congo Basin strain DNA. J Virol Methods. 2010;169:223–7. 10.1016/j.jviromet.2010.07.012 20643162 PMC9628942

[6] Karan A, Styczynski AR, Huang C, Sahoo MK, Srinivasan K, Pinsky BA, et al. Human Monkeypox without viral prodrome or sexual exposure, California, USA, 2022. Emerg Infect Dis. 2022;28:2121–3. 10.3201/eid2810.221191 35971952 PMC9514362

[7] Ferré VM, Bachelard A, Zaidi M, Armand-Lefevre L, Descamps D, Charpentier C, et al. Detection of monkeypox virus in anorectal swabs from asymptomatic men who have sex with men in a sexually transmitted infection screening program in Paris, France. Ann Intern Med. 2022;175:1491–2. 10.7326/M22-2183 35969863

[8] De Baetselier I, Van Dijck C, Kenyon C, Coppens J, Michiels J, de Block T, et al.; ITM Monkeypox study group. Retrospective detection of asymptomatic monkeypox virus infections among male sexual health clinic attendees in Belgium. Nat Med. 2022;28:2288–92. 10.1038/s41591-022-02004-w 35961373 PMC9671802

[9] US Food and Drug Administration. FDA monkeypox response: FDA’s role in mpox preparedness and response, and information about mpox (formerly referred to as monkeypox) [cited 2023 Jan 7]. https://www.fda.gov/emergency-preparedness-and-response/mcm-issues/fda-monkeypox-response

